# An inclusive economy dataset for wards in Great Britain using administrative and synthetic data sources

**DOI:** 10.1038/s41597-025-05502-x

**Published:** 2025-07-15

**Authors:** Hugh P. Rice, Andreas Höhn, Petra Meier, Alison Heppenstall, Nik Lomax

**Affiliations:** 1 Institute for Spatial Data Science, School of Geography, University of Leeds, Leeds, LS2 9JT UK; 2 School of Health and Wellbeing, University of Glasgow, Glasgow, G12 8TB UK; 3 School of Geography and Sustainable Development, University of St. Andrews, St Andrews, KY16 9AL UK; 4 School of Social and Political Sciences, University of Glasgow, Glasgow, G12 8TB UK; 5 Turing Institute, London, NW1 2DB UK

**Keywords:** Decision making, Geography, Public health

## Abstract

To address the scarcity of small-area datasets focused on economic inclusion, we created a harmonised dataset describing the extent and enablers of economic inclusion in Great Britain. The result, the SIPHER (Systems Science in Public Health and Health Economics Research) Inclusive Economy (Ward Level) dataset, consists of 13 indicators describing economic inclusion at electoral ward level (*N* = 7,973 of 8,020 wards, 2022 boundaries), for 2019–2021. The dataset was curated based on administrative statistics (mostly open-source) and the SIPHER Synthetic Population, a validated, survey-based, full-scale synthetic population dataset derived from the UK Household Longitudinal Study (UKHLS): Understanding Society, and aggregate-level population statistics. The dataset also includes summary measures of population health – age-standardised Short Form Health Survey (SF-12) mental and physical health component scores – and supplementary demographic indicators describing the population structure. For validation, a range of comparisons against deprivation indices and other data provide strong evidence of the dataset’s added value and utility for applications in research and policy requiring high-quality estimates at a granular spatial resolution.

## Background & Summary

### Granular spatial data for policy planning and research

Spatial data at a granular scale are essential for policy planning as a greater spatial resolution gives decision-makers more fine-grained information. Here, this is specifically relevant for two tasks. Firstly, for the design, operationalisation and implementation of localised policies (e.g. identifying vulnerable populations or priority sites for interventions), and secondly for an assessment of likely effects of national or regional policies at the local level prior to implementation, especially regarding unintended inequalities-related consequences. As Deas, *et al*.^1^ noted, “local geographies [are] central to challenging the previously dominant [agglomeration-based] model and promoting new thinking around inclusive growth and inclusive economies” (p. 179). In light of a renewed political consensus around expanding sub-regional devolution in the UK^2^, opportunities available to policy-makers in developing post-Covid-19 recovery strategies^3^ and the cost-of-living crisis^4^, there continues to be a major focus on the importance of economic inclusion. One central argument is that a decrease in economic inclusion might have contributed to the stalling of population health improvements that has been observed across many high-income countries, including England, Scotland and Wales^5,6^.

Specifically for the UK, there is a significant lack of readily available resources that bring together key measures of economic inclusion. Data scarcity is particularly acute for Northern Ireland, and so we restrict our focus to Great Britain (GB) hereafter, i.e. England, Scotland and Wales. Currently, datasets capture the nations of GB in isolation and focus primarily on the concept of deprivation. For example, datasets of the Indices of Multiple Deprivation (hereafter IMD collectively) are produced at intervals of a few years separately for England: English Indices of Deprivation (EID, 2019), Scotland: Scottish Index of Multiple Deprivation (SIMD, 2020) and Wales: Welsh Index of Multiple Deprivation (WIMD, 2019). Although some methods for comparison between nations have been developed^7^, no single, harmonised version of the IMD datasets exists. In addition, there are at least three significant differences between national indices that make harmonisation difficult. Specifically, (a) the domains themselves in each dataset differ; for example, the “living environment” domain in the EID is broadly equivalent to the “physical environment” domain in the WIMD, but no equivalent exists in the SIMD; (b) the indices overall are calculated using different weights per domain, and (c) datasets for each nation are not updated simultaneously. Given these limitations, an all-UK or all-GB dataset covering multiple aspects of economic inclusion would be of high utility for understanding the relative effectiveness of policy across different geographical areas, their strengths and weaknesses with respect to specific indicators/domains, and how they develop over time.

Our dataset is not presented as an alternative to national-level IMD datasets, which are targeted at specific domains such as health and community safety. It is instead intended to satisfy three requirements, none of which are covered by eight other indicator datasets reviewed in the technical report on which this dataset is based^8^, namely that: (a) the dataset is harmonised to cover all of GB rather than specific cities or devolved nations, (b) it is focused on economic inclusion rather than other concepts, and (c) it is available at the granular level of electoral wards rather than larger areas.

### Building economic inclusion into political strategies

Particularly since the Covid-19 pandemic, there has been an increased focus on the importance of economic inclusion and its embedding into policy-making^3^. However, there is currently no universally agreed definition of what constitutes an inclusive economy^9^. In the absence of a universal definition and commensurate metrics, translating commitment amongst policy-makers into action, and evaluating the success of such interventions, remains challenging.

In addition to the existence of heterogeneous definitions, there is the competing concept of inclusive growth^9,10^. While inclusive economic growth is often invoked as an enabler of social participation^11^, economic inclusion is a potent driver of improved population health and reduced health inequalities^12^. For this dataset, we focus on the concept of economic inclusion only. In their reviews of the inclusive economy literature, Macintyre, *et al*.^13^ and Shipton, *et al*.^9^ describe the following characteristics of an inclusive economy:

- *Deliberate design* of an economy to be inclusive, i.e. through regulations, policies and governance that allow the extent to which equity is delivered to be assessed.
- *Equitable distribution* of the benefits of such an economy through, for example, goods and service, health and power.
- *Equitable access* to the resources necessary for economic participation, such as good health, social support and education.

Where inclusive economy and wellbeing economy principles meet, an additional criterion concerns the operation of the economy within *planetary resources*; that is, “the full environmental costs of economic activity are included … in the cost of production and distribution of goods and services” ^9^ p. 1131. For this dataset, this characteristic was not operationalised, as the focus was on equity rather than broader concepts such as social justice, de-growth and the climate crisis. However, there is ongoing work to expand into these areas through the GALLANT research consortium (GALLANT - Glasgow as a Living Lab Accelerating Novel Transformation)^14^.

### Health and wellbeing impacts of economic inclusion

As to the motivation behind embedding economic inclusion in policy implementation, there is a large and growing body of research concerned with the bi-directional relationships between economic inclusion, socioeconomic status, and various aspects of physical and mental wellbeing, including health and health inequality. Bambra, *et al*.^15^ noted that “health follows a social gradient: better health with increasing socioeconomic position” (p. 284). This observation is consistently reflected across different health outcomes. For example, life expectancy tends to be lowest while lifespan variation tends to be highest among the most deprived in society^16,17^. Very similar patterns have been reported for other indicators of social position such as educational attainment^18^, and occupational class^19^. Equally strong relationships have been found between various aspects of socioeconomic inequality and wellbeing, such as that between income and mental health^20^, all-cause mortality^21^ and adolescent health^22^. McCartney, *et al*.^23^ noted that “[p]opulation health … is largely socially determined” (p. 1) and that socioeconomic inequality is the principal cause of health inequalities.

Alongside the relationship between deprivation and life expectancy/lifespan variation, recent studies have increasingly drawn attention to the role of economic inclusion for explaining area-level health inequalities. In a recent study, the SIPHER Inclusive Economy (Local Authority Level) dataset was presented in the form of 13 indicators capturing aspects of the inclusive economy, alongside demographic and wellbeing indicators^24^. That dataset was used by Höhn, *et al*.^25^ to investigate the relationship between inclusive economy indicators and quality-adjusted life expectancy (QALE) across local authority in GB. It was found that aspects of economic inclusion accounts for more than half of the variation in QALE amongst local authorities.

In their review of the effects of policy interventions on inclusive economy outcomes, Macintyre, *et al*.^13^ focused on two outcome domains: (a) the distribution of benefits arising from economic inclusion to the economy, and (b) access to resources required for economic participation. They found that the efficacy of interventions largely considered intermediate interventions – such as moving people into employment – leaving substantial evidence gaps around the efficacy of structural-level economic reform designed to embed inclusion at the point of action. They also found that most reviews were concerned with the targeting of policies to, and their effect on, specific disadvantaged groups, rather than with population-wide interventions. Bambra, *et al*.^15^ noted that it appears that “not only is the public health systematic review evidence base weak in terms of how to tackle the social determinants [of health and health inequalities], but that there are specific areas that appear especially sparsely populated” (p. 290), including policy effects on health and health inequalities.

### Creating a small area-level dataset of inclusive economy indicators

With these research gaps in mind – specifically the sparsity of small-area data related to health and health inequalities – we present this dataset of inclusive economy indicators at electoral ward level. The aims of our dataset are to explore the link between economic inclusion and health indicators at ward level in GB, and to provide a readily available collection of indicators that captures the core concept of economic inclusion. If used in different analyses, our dataset has the potential to provide valuable insights into local and regional inequalities and highlight opportunities for policy interventions.

In their post-Covid-19 recovery strategy, Public Health England^26^ emphasised the need for equitable access to local services and area-level action on economic inclusion and sustainability as means for reducing health inequalities, particularly when targeted at disadvantaged groups. Small-area classifications can be used to both explain variation in outcomes between and within larger areas, and inform policy as a result, and have found use in a broad range of applications^27^. For example, Clark, *et al*.^28^ used data from individual body-worn accelerometers to assess the effect of severe illness and mortality due to Covid-19 infection, concluding that health outcomes thus quantified provide an opportunity to inform personalised policy recommendations. Grubesic, *et al*.^29^ investigated the county-level distribution of diabetes prevalence by lifestyle classification in the United States. The authors noted that such small-area data can be used to identify at-risk populations and target interventions. In their study of the link between geodemographic indicators and prevalence of limiting long-term illness, Moon, *et al*.^30^ emphasised the importance of small-area data for health service planning, health inequality measurement and care quality assessment. Similarly, Abbas, *et al*.^31^ noted the utility of such small-area data for informing health service planning: they give better insights into health inequalities as they allow for assessment of outcomes against multiple variables, rather than against univariate data across domains such as deciles or quintiles of deprivation, for example.

Our dataset was conceptualised by the SIPHER Consortium^32^. It allows the relationship between economic inclusion and health outcomes to be compared across small geographic areas through a meaningful collection of indicators that is, nevertheless, concise enough to avoid unnecessary complexity. The conceptualisation of economic inclusion captured in this dataset is the outcome of an iterative review and consultation process through which the indicators were selected, involving SIPHER researchers, SIPHER policy partners, a national topic advisory panel and community panels consisting of citizens with lived experience of economic exclusion and/or poor health. Full details of the conceptualisation and consultation process is given in a previous technical report^8^.

The definitions of the inclusive economy indicators presented here match as closely as possible those in the SIPHER Inclusive Economy (Local Authority Level) dataset^24^. Based on the same technical report, this dataset captures the concept of economic inclusion for the 363 lower-tier local authority districts in GB. The indicators and the metrics used for each dataset are given in Table 1 (inclusive economy indicators, outcomes/category A), Table 2 (inclusive economy indicators, wider outcomes and enablers/category B) and Table 3 (demographic and wellbeing indicators).

**Table 1:** Specification of indicator data sources and variables, inclusive economy indicators, economic outcomes category (A).

ID	Descriptor	Metric(s)	Data source(s); variable(s) used	Variable definitions and specifications
1A*	Participation in paid employment	Percentage of working-age people who are employed	UKHLS^2^; *dvage*, *employ*	*Definitions*: “The age of the respondent at last birthday” (*dvage*); “[Are you] in paid employment?” (*employ*). *Numerator*: All in denominator who are employed (*employ* = 1). *Denominator*: All in age range 16–64 (*dvage*).
2A*	Involuntary exclusion from the labour market	Share of working-age people who are long-term unemployed or inactive due to ill health or disability	UKHLS^2^; *jbstat*	*Definition*: “[Which category] best describes your current employment situation?“. *Numerator*: All in *jbstat* = 8 (long-term sick or disabled). *Denominator*: All with valid *jbstat* values (*jbstat* > 0).
3A	Wealth inequality	Ratio of median house prices in most/least expensive areas	ONS here (England and Wales)^1^; SG here (Scotland)^1^	Max/min median house prices by LSOA within wards.
4A*	Earnings inequality	Ratio of weekly earnings (residents in FT work), 80^th^/20^th^ percentiles	UKHLS^2^; *paygu_dv*, *jbstat*, *jbft_dv*	*Definition*: “Usual gross pay per month” (*paygu_dv*); as Indicator 2A (*jbstat*); “Employed full time (i.e., greater [than] 30 hours per week)” (*jbft_dv*). *Specification*: *paygu_dv* > 0, ratio of 80^th^/20^th^ percentiles; full-time employees only (*jbstat* = 2; *jbft_dv* = 1).
5A*	Poverty	Percentage of children living in low-income households	DWP (via Gov.UK)^1^ here	Relative poverty before housing costs (BHC) is measure of low income used, i.e. households for which income falls below 60% of national median; details here. After housing costs (AHC) data, as used in local authority-level dataset, not available at ward level.
6A*	Decent pay	Proportion of employee jobs that are paid at or above the Real Living Wage	UKHLS^2^; *paygu_dv*, *jbhrs*, *jbstat*, *dvage*	*Definitions*: As Indicator 4A (*paygu_dv*); “[H]ours normally worked per week“ (*jbhrs*), as Indicator 2A (*jbstat*); as Indicator 1A (*dvage*). *Numerator*: Those in denominator with hourly earnings (derived from *paygu_dv* and *jbhrs*) above Real Living Wage (18 + only, from Living Wage Foundation here; different rates applied for London and non-London). *Demoninator*: Employees (*jbstat* = 2) with non-zero income (*paygu_dv* > 0) aged 18 or over (*dvage* ≥ 18).
7A*	Job security	Share of employees in permanent work	UKHLS^2^; *jbstat*, *jbterm1*	*Definition*: As Indicator 2A (*jbstat*); “Current job: permanent or temporary” (*jbterm1*). *Numerator*: Employees (*jbstat* = 2) in permanent jobs (*jbterm1* = 1). *Denominator*: Employees in permanent or non-permanent jobs (*jbterm1* in [1, 2]).

UKHLS = Understanding Society/SIPHER Synthetic Population, ONS/Nomis = Office for National Statistics/website, SG = Scottish Government, DWP = Department for Work and Pensions.

*Data source or definition of variable/metric differs from that in local authority-level dataset.

^1^Input data are included with data package at data repository^35^.

^2^Input data are not included with data package at data repository^35^; see instructions in user guide there.

**Table 2:** Specification of indicator data sources and variables, inclusive economy indicators, wider outcomes and enablers category (B).

1B*	Skills and qualifications	Percentage of adults aged 20–49 with a Level 2 or higher NVQ qualification	UKHLS^2^; *hiqual_dv*, *dvage*	*Definitions*: “Current status highest educational or vocational qualification” (*hiqual_dv*); as Indicator 1A (*dvage*). *Numerator*: All with GCSE-level or higher qualification (*hiqual_dv* in [1, 2, 3, 4]) in age range (20 ≤ *dvage* ≤ 49). *Denominator*: All in numerator with valid values (*hiqual_dv* > 0).
2B	Digital connectivity	Engagement with digital at LSOA level based on Internet User Classification (IUC)	CDRC^2^ here	Proportion of LSOAs by ward classed as digitally disengaged, i.e. in categories 7 (“Passive and Uncommitted Users”), 9 (“Settled Offline Communities”) or 10 (“e-Withdrawn”).
3B	Physical connectivity	Public transport accessibility measure	NAO data here (England)^1^; WABI here (Wales)^2^; SABI here (Scotland)^1^	Different specification for each nation: England is proportion of LSOAs by ward with score in [2, 3, 4, 5, 6, 7], Wales is those with decimal score below median, Scotland is those in lower five deciles (weekday data).
4B*	Housing affordability	Ratio of median house prices to median (workplace) earnings	House price data: as Indicator 3A (i.e. ONS)^1^; earnings from UKHLS^2^; *fimnlabgrs_dv*	*Definition*: “Total monthly gross personal labour income” (*fimnlabgrs_dv*)*. Specification*: *fimnlabgrs_dv* > 0.
5B*	Cost of living	Fuel poor households	UKHLS^2^; *hheat*	*Definition*: “In winter, are you able to keep this accommodation warm enough?” (*hheat*). *Numerator*: All answering “no” (*hheat* = 2). *Denominator*: All answering “yes” or “no” (*hheat* = 1 or 2).
6B	Inclusion in decision-making	Voter turnout in local elections	EC^1^ (website and communication with authors)	Voter turnout by electoral ward.

UKHLS = Understanding Society/SIPHER Synthetic Population, ONS/Nomis = Office for National Statistics/website, CDRC = Consumer Data Research Centre, NAO = National Audit Office, EC = Electoral Commission.

*Data source or definition of variable/metric differs from that in local authority-level dataset.

^1^Input data are included with data package at data repository^35^.

^2^Input data are not included with data package at data repository^35^; see instructions in user guide there.

**Table 3:** Specification of indicator data sources and variables, demographic and wellbeing indicators.

TDR	Total dependency ratio	Ratio of dependants to working-age adults	UKHLS^2^; *dvage*	*Definition*: (*N*(0–15) + *N*(65+) / *N*(16–64) x 100). As Indicator 1A (*dvage*).
SR	Sex ratio	Ratio of number of males to females	UKHLS^2^; *sex*	*Definition*: *N*(*sex* = 1, i.e. “male”) / *N*(*sex* = 2, i.e. “female”).
SF-12	Wellbeing measure	SF-12 mental and physical component scores (MCS, PCS), age-standardised	UKHLS^2^; *sf12mcs_dv*, *sf12pcs_dv*	*Definition*: Individual-level SF-12 scores for mental (MCS) and physical (PCS) health were derived via scoring algorithm by UKHLS survey team, based on range of survey questions capturing different domains of health. Resulting summary score for individuals represents continuous scale with range of 0 (low functioning) to 100 (high functioning). Summary score for entire electoral ward was created via age-standardisation of age-specific SF-12 summary scores, based on EU Standard Population 2013 (i.e. same approach as for deriving age-standardised mortality rates).

UKHLS = Understanding Society/SIPHER Synthetic Population.

^1^Input data are included with data package at data repository^35^.

^2^Input data are not included with data package at data repository^35^; see instructions in user guide there.

Our dataset presented in this paper reflects the level of electoral wards in GB. Wards are substantially smaller than local authorities, thereby offering greater spatial resolution. In England, Scotland and Wales, the mean population size of a ward in 2021 was *N* = 8,236, *N* = 15,436 and *N* = 4,215 individuals (2022 ward definitions), respectively. At even smaller geographical units, e.g. Lower Layer Super Output Areas (England and Wales, hereafter LSOAs) or Data Zones (Scotland, hereafter DZs), data sparsity becomes an issue, particularly when working with non-synthetic sources of data (e.g. area-linked surveys). Electoral wards therefore represent a “sweet spot” in terms of data availability and utility for local policy decision-making.

Our dataset allows statistics to be tracked over a three-year period (2019–2021) and is limited to GB rather than the whole UK, as data for Northern Ireland were not available for several indicators. This limitation, along with the diversity of data sources used, illustrates the complex landscape of the UK’s decentralised and devolved statistical bodies, and some remarks on updateability of the dataset are given later.

## Methods

### Overview of dataset and its development

The dataset covers 7,973 of the 8,020 electoral wards in GB, i.e. England, Wales, and Scotland; 47 wards (0.59% of the total) are not present, and these are in 17 local authorities, of which 31 of the missing wards were in three local authorities: the City of London (20 wards), Gwynedd (7) and the Isles of Scilly (4). Those 47 wards are not present either because (a) they are “subthreshold” wards whose population or household counts are too low to remain non-disclosive (see the ONS Postcode Directory user guide^33^), or (b) they don’t contain a 2011 LSOA population-weighted centroid (confirmed by the authors by communication with the Office for National Statistics, hereafter ONS).

The development process of our dataset consisted of the following stages. Firstly, a hybrid data sourcing strategy was used, where (a) open/bespoke sources were used wherever possible, and (b) the SIPHER Synthetic Population was used when other data sources weren’t available. Secondly, data for all indicators were standardised and expressed in terms of 2022 electoral ward definitions, where it was necessary to aggregate from smaller areas (LSOAs and DZs) in some cases. Lastly, once the final dataset had been assembled at ward level, missing data were imputed. More details of each stage of the development process are given in the subsections that follow.

### Strategy for sourcing and processing of input data

A hybrid data sourcing strategy was employed to construct the dataset. Data were gathered from open sources (e.g. national statistical agencies), bespoke sources (via academic researchers) and synthetic data sources – specifically, the SIPHER Synthetic Population (2020)^34^. An overview of all data sources is given in Tables 1–3. The hybrid data sourcing approach was chosen so that the dataset would match as closely as possible the data sources used for the accompanying local authority-level dataset^24^ in the interests of comparability, and so that any data not available at electoral ward level could be replaced with synthetic population data. Open-source or bespoke data were used where available, and synthetic population data were used otherwise.

**Fig. 1:**
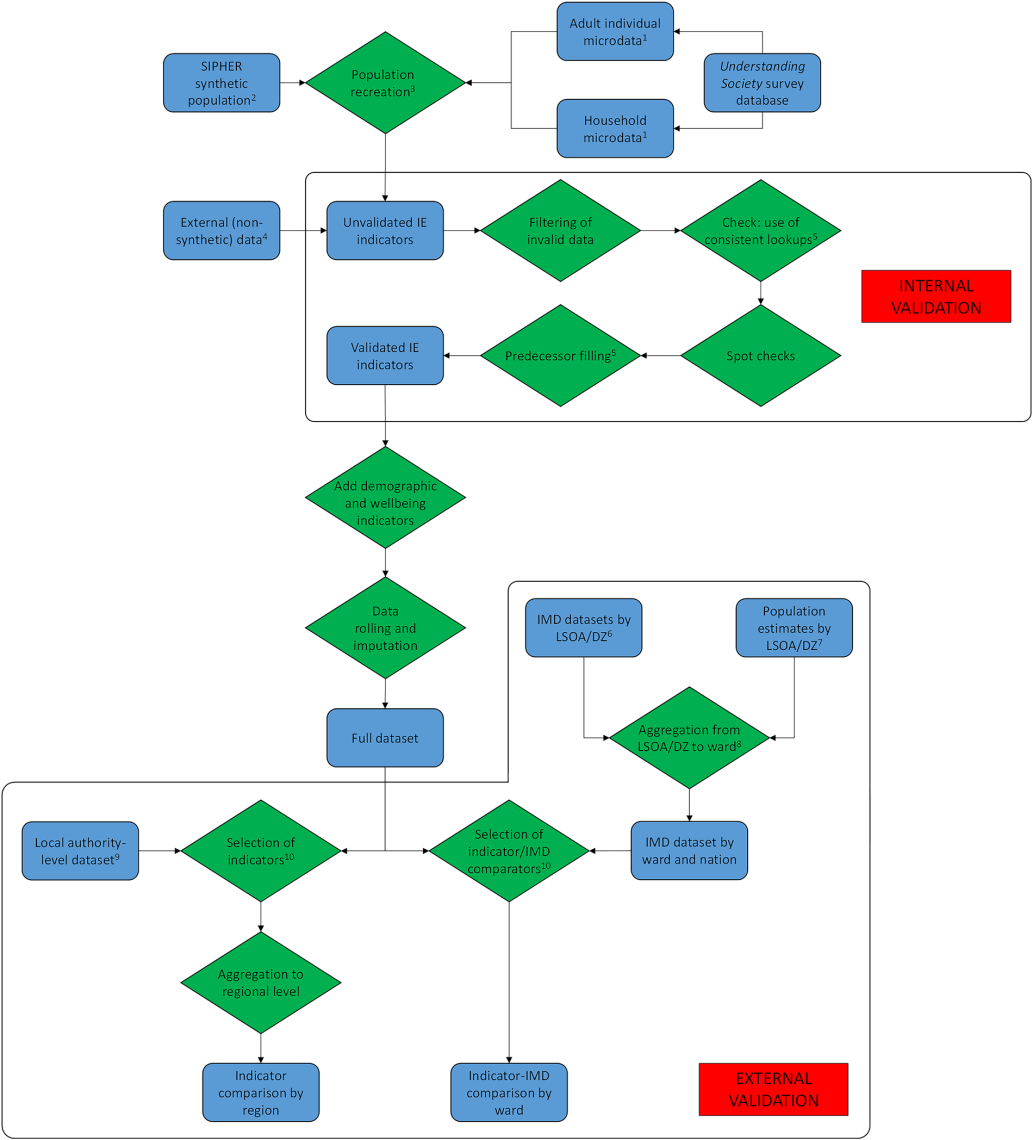
Flowchart of overall data sourcing, processing and validation. 1: Selecting for variables of interest, i.e. inclusive economy indicators, SR, TDR and SF-12. 2: At LSOA/DZ level (EW/S); aggregated to ward later. 3: Duplication of individuals in synthetic population to create ward-level population. 4: Certain indicators only; see main text. 5: All external (i.e. non-synthetic) indicator data sources given in Tables 1, 3. 6: Separate sources for E (2019), S (2020) and W (2019), all by LSOA/DZ (EW/S). 7: Separate sources for EW and S, duplicated 2020 data to 2021 for EW; see main text. 8: Used GLA methodology for aggregating by rank^60^; see main text. 9: IMD and external comparator sets given in main text. 10: See main text for discussion of ward-level to LA-level comparison.

A flowchart of the overall data sourcing, processing and validation strategy is shown in Fig. 1. For each indicator, the best available data source was chosen, where “best” embodies the balance between data coverage/availability, and closeness to the concept behind the indicator, as originally described in the technical report on which both this and the previously published local authority-level dataset are based^8^. Those indicators for which definitions or data sources differ from those in the local authority-level dataset are marked with an asterisk in Tables 1, 2, and further details are given there.

Of the 13 inclusive economy indicators presented, five indicators use external (i.e. non-synthetic) data only (Indicators 3A, 5A, 2B, 3B and 6B), of which two indicators were not open source (2B and 3B). Seven indicators use data from the SIPHER Synthetic Population only (1A, 2A, 4A, 6A, 7A, 1B and 5B). Lastly, one indicator uses a combination of synthetic and non-synthetic data (4B). The variables were processed such that all were in a common format, ready for data imputation, which required pre-processing in the form of filling/rolling of data. This stage is summarised in Table 4 and described in detail the “Imputation of missing data” section.

**Table 4:** Data validation and development summary per indicator.

ID	Descriptor	Validation summary (missingness for 2020 unless otherwise stated)**	Years of input data available; years computed/presented + rolling rules for imputation (if any)
1A*	Participation in paid employment	Missingness: 0.59%.	1991–2021 (*dvage*), 2009–2021 (*employ*); 2019–2021
2A*	Involuntary exclusion from the labour market	Missingness: 0.59%.	1991–2021 (*jbstat*); 2019–2021
3A	Wealth inequality	Missingness: 0.59%. Uses LSOA 2011 to ward 2022 map.	1995–2021 (house price data); 2019–2021
4A*	Earnings inequality	Missingness: 0.59%.	1991–2021 (*fimnlabgrs_dv*); 2019–2021
5A	Poverty	Missingness: 0.26% (City of London and other sparsely inhabited areas). Uses ward 2021 to ward 2022 map.	2015–2022 (relative poverty data); 2019–2021
6A*	Decent pay	Missingness: 0.59%.	1991–2021 (*fimnlabgrs_dv*), 1991–2021 (*jbhrs*), 1991–2021 (*jbstat*); 2019–2021
7A*	Job security	Missingness: 0.59%.	1999–2021 (*jbterm1*); 2019–2021
1B*	Skills and qualifications	Missingness: 0.59%.	1991–2021 (*hiqual_dv*); 2019–2021
2B	Digital connectivity	Missingness: 0.59%. Uses LSOA 2011 to ward 2022 map.	2018 only (IUC data); 2018 values carried forward to 2019–2021
3B	Physical connectivity	Missingness: 0.59%. Uses LSOA 2011 to ward 2022 map.	2017 (bus accessibility data for England), 2017 and 2019 (Scotland), 2019 and 2022 (Wales); most recent data for England and Scotland carried forward, Wales data for 2022 rolled backward
4B*	Housing affordability	*For house price data only (as earnings data are from UKHLS):* Missingness: 0.63% (27.3% before filling). Uses ward 2020 to ward 2022 change dictionary for predecessor filling.	1995–2022 (house price data), 1991–2021 (*fimnlabgrs_dv*); 2019–2021
5B*	Cost of living	Missingness: 0.59%.	2009–2020 (*hheat*); 2019–2021
6B	Inclusion in decision-making	Missingness: 1.55% (3.94% before predecessor filling, then 2.88% before filling with manually added data). Wards matched by name, as errors present in ward codes. Uses 2016 to 2022 ward change dictionary for predecessor filling. Also 200 + wards with missing data, some of which found online and added manually. Others missing as councillors were elected unopposed.	2016–2022, except 2020 as no local elections held due to Covid pandemic, but data missing for many areas and years, as local elections not held in every nation every year; 2020 data for England mostly available, but rolled forward from previous years when necessary, 2017 data for Wales and Scotland rolled forward to 2019–2021

Missingness reflects the level of missing information after application of rolling rules, and before the usage of multiple imputation. UKHLS = Understanding Society, EC = Electoral Commission.

*UKHLS variables used partly (Indicator 4B) or wholly for these indicators, i.e. SIPHER Synthetic Population merged with UKHLS.

**Missingness of 0.59% observed for multiple indicators. This is result of 47 of 7,973 wards being excluded as lacking population centroid, or due to disclosivity; see “Methods” section.

The codebase was written in Python and R using the Anaconda/Miniconda environment manager, and the Pycharm (Python) and RStudio (R) integrated development environments, respectively. Raw input data used during creation of the dataset come from multiple sources, several of which are not open-source and could not be included in the dataset for reasons of size or confidentiality. Specifically, these were: (a) the Understanding Society survey data (UK Data Service: SN6614, general end-user license), (b) the SIPHER Synthetic Population (UK Data Service: SN9277, general end-user license), (c) the ONS change history, (d) input data for Indicators 2B (digital connectivity, via CDRC) and (e) input data for 3B (physical connectivity data for Wales via Wiserd), all of which must be sought by the user if they wish to recreate the dataset. Details of how to obtain these data are given in the user guide at the OSF data repository^35^.

### Inclusive economy indicators obtained from non-synthetic data sources

Within the hybrid data sourcing methodology used to construct the dataset, five inclusive economy indicators were derived partly or wholly from non-synthetic (i.e. external) sources. As an example, there follows a description of our indicator development process for Indicator 6B (indicator: inclusion in decision-making; metric: voter turnout at local elections). When screening data raw input data, it became clear that the ONS codes for electoral wards in some of the records provided by the Electoral Commission contained errors accrued during compilation (confirmed by communication with the authors). As a result, a strategy was implemented to match found records to reference records by electoral ward names alone, rather than ward codes. A brief description is given here, and more details are available from the authors on request. The central challenge to be overcome was that ward names contain a greater range of potential disparities than do ward codes, and the goal of the matching strategy was to minimise missingness, which was quantified at each stage and is summarised in Table 4.

Our matching strategy for Indicator 6B is an example of the linear sum assignment problem^36^ for which solutions exist^37^. The basis of this problem is to find the optimal set of matches between two groups of items – in this case reference and found ward names – where “optimal” means the set of matches that maximises the degree of similarity between records in some sense. Here, similarity was quantified using a metric based on the Levenshtein distance^38^, where the similarity between record pairs (specifically ward names) took values in the range 0 ≤ *s* ≤ 1.

For each year of available data, firstly, a set of matches was sought using the matching strategy described, with a similarity threshold applied, such that matches were only made if the similarity between candidate pairs exceeded the threshold. A threshold value of *t* = 0.6 was determined using 2016 data such that (a) the number of correct matches was maximised and (b) the number of incorrect matches was minimised; it was then applied to all years of data (2016–2022). To then account for coarser disparities between records that could not be matched in this way, matches were sought between any ward pairs for which each reference ward name had only one candidate found ward name that also contained it.

The matching process overall was performed within each local authority area in turn, as there were many wards with duplicate names across multiple local authorities (e.g. “Castle”: 63 instances, “Park”: 51 instances, etc.) and because this method significantly reduced the computational complexity of the matching process.

### Using the SIPHER synthetic population

Around half of all indicators in the dataset were derived from the SIPHER Synthetic Population, a full-scale digital twin of the GB adult population aged 16 years and older. Its creation and the validation of the underlying methodology is described elsewhere in full detail^34,39^. The SIPHER Synthetic Population, accompanied by a rich user guide and validation report, is available for full independent use via the UK Data Service^34^ as an area-level linkage file for the UKHLS main survey dataset.

The SIPHER Synthetic Population was constructed from two sources: UKHLS (the source of the individuals in the dataset) and administrative population statistics data for small areas (from which constraints data were derived). A detailed description of the eight constraints (e.g. age/sex, ethnicity) and how they were used to construct the synthetic population can be found in the user guide^34^, and supplementary material^40^. While data from the UKHLS survey was drawn from the period 2019–2021, the constraints data are mainly drawn from the 2011 UK Census and 2020 population estimates.

The synthetic population was created with the Flexible Modelling Framework (FMF) software^41,42^, which employs simulated annealing^43,44^, a form of combinatorial optimisation. The aim of this process is to find the combination of individuals from a sample population (here UKHLS) such that observed aggregated population characteristics from known sources (here constraints data) for each small area (here LSOAs and DZs) are matched. That is, for each area, a proposed population is selected randomly, then an incremental random draw of individuals is selected and is added to the population by replacement if doing so improves the correspondence with the constraints. Individuals are exchanged in this way iteratively until the correspondence is within a specified tolerance. As the algorithm operates through replacement of individuals, the same individual can appear multiple times within the population.

UKHLS spans the period 1991 to the present, and the latest wave of data is available for 2022–2024 (wave 14 or “n”)^45^. Hereafter, each UKHLS wave is identified by its central year, e.g. wave 11/”k” as “2020”. Since its inception, UKHLS has contained responses from around 100,000 people across 40,000 households. In the absence of a comprehensive population-based register reflecting the UK population – as in the Nordic countries, for example^46^ – the power of UKHLS lies in its ability to capture a wealth of life domains longitudinally, reflecting a nationally representative sample of households and individuals. The main survey of UKHLS consists of a highly detailed set of responses to survey questions. All responses are coded into variables at various levels, e.g. individual or household. Since its launch, UKHLS has been used in a wide range of socioeconomic and demographic research, resulting in over 4,300 publications to date^47–49^.

The SIPHER Synthetic Population^34,39^ can be understood as a synthesised version of the UKHLS survey at the level of LSOAs/DZs. LSOAs are small administrative units in England and Wales containing approximately 1,500 inhabitants, and DZs are the equivalent units in Scotland that capture around 500–1,000 inhabitants. Although the process of creating the synthetic population is not the focus of this study, a short summary follows.

To obtain the synthetic population-derived indicators presented in this paper (i.e. indicators 1A, 2A, 4A, 6A, 7A, 1B, 4B and 5B) the SIPHER Synthetic Population was populated with individual- and household-level data from UKHLS to compute the metrics used for several indicators (see Tables 1, 3) via the following process.

1. Obtain the SIPHER Synthetic Population for GB. The dataset has the form of persistent (i.e. cross-wave) personal identifiers (*pidp*) and the respective LSOA/DZ area code for each *pidp*.
2. Link the SIPHER Synthetic Population with UKHLS survey data at individual and household level based on *pidp*.
3. Filter data according to valid and invalid/missing values, and correct data as necessary, to ensure efficient computation of final indicators; see the “*Technical Validation”* section for more details.
4. Aggregate from LSOA level, at which the SIPHER Synthetic Population was constructed, to electoral ward level, at which this dataset is presented.

As UKHLS is a panel survey, the personal identifier *pidp* is persistent across multiple waves. This means that it is possible to compute the metrics used in this dataset for any chosen year, and not only the year for which the synthetic population was computed (i.e. 2020). However, the greater the time difference between the reference year (i.e. that for which the synthetic population was calculated, here 2020) and the year of interest, the fewer individuals present in the synthetic population will also be present in the UKHLS survey in a chosen year. This is due to year-on-year mortality and attrition among individuals, and because new participants can join the survey.

As a result of the foregoing considerations, we sought a balance between chronological coverage and representativeness with respect to the issue of participants being present across multiple waves, when constructing the dataset. Therefore, we obtained indicators from this synthetic source for 2020 (reference year of the SIPHER Synthetic Population), and one year either side. The proportion of individuals present in the synthetic population that were also present in those years of data was: 91.4% (2019), 100% (2020, by definition) and 85.2% (2021). These figures closely match the year-on-year participation rate of 90% stated in the UKHLS documentation^50^. However, due to the underlying cross-sectional format of the SIPHER Synthetic Population, longitudinal patterns among those indicators obtained (fully or in part) from the synthetic source should be interpreted with caution as the attrition described earlier might have contributed to trends in the data.

Further to this, it is instructive to draw a distinction between constraint variables and non-constraint variables – i.e. those variables that were used in the creation of the synthetic population and those that weren’t. Variables in the former category are known to match small-area statistics very closely – in our case LSOAs and DZs – as this is, of course, the purpose of the synthetic population creation process. Variables of the latter type, however, were not aligned to match small-area statistics. The variables derived from the SIPHER Synthetic Population used in this study fall into both categories. As a result, no direct statistical validation of the non-constraint variables used here (e.g. *hheat*, *paygu_dv*) was possible - since no benchmark data for direct comparison exist, to our knowledge. However, using the SIPHER Synthetic Population to fill gaps around health data and the socioeconomic environment in this way is entirely within its recommended range of applications – in line with existing publications^34,51^.

Although they can be used to fill data gaps, synthetic population datasets come with limitations. Unlike real data, which reflect information collected in real-world settings, synthetic data are the result of a statistical creation process. This links the quality of synthetic information directly with the quality of the creation and validation process. At the same time, estimates obtained from synthetic sources can, in some circumstances, provide more reliable estimates than real-world data – particularly in cases where real data suffer from small sample sizes, non-representative sampling or drop-out. In this study, all indicators derived from the SIPHER Synthetic Population were not available from real-world sources at the required granular scale.

For our purposes, we used the income variables *fimnlabgrs_dv* (total monthly gross personal labour income) and *paygu_dv* (usual gross pay per month) – specifically for Indicators 4A (earnings inequality), 6A (decent pay) and 4B (housing affordability). To avoid numerical problems when calculating the corresponding metrics, it was necessary to exclude any individuals with an income code of zero or below zero (typically “–8”/”-9” indicating a missing or inapplicable value). For example, the metric for Indicator 4A consists of the ratio of the 80^th^ to 20^th^ income percentiles, which generally could not be calculated unless zero and below-zero incomes were excluded (e.g. 43% of individuals in 2019 data had such zero/below-zero income, i.e. wave 10/“j”).

### Harmonisation of data to 2022 electoral wards and predecessor filling

We harmonised data for all indicators to the 2022 definitions of GB electoral wards. Electoral ward definitions generally change every year, to a greater or lesser degree. Although some changes were simple (for example, changes to names and/or codes only), others were more complex (i.e. changes to geographical boundaries, often including aggregations or divisions of previous wards, which in turn incorporate changes to names and/or codes). LSOA definitions change less frequently, most recently in 2001, 2011 and 2021. DZ definitions changed in 2001, 2011 and 2022. These changes had to be accounted for in the harmonisation process, as input data were found in the format of both 2011 and 2021 LSOAs, and 2011 DZs (no relevant 2022 DZ-based data were available at the point of creation).

Any mappings between different LSOA/DZ and ward definitions that were performed are given per indicator in Table 4. For example, the input data for Indicator 5A (poverty) were found by 2011 LSOAs/DZs. It was therefore necessary to aggregate to 2022 wards. For Indicator 6B, it was necessary to convert all data to 2022 wards for the period 2016 to 2021, where ward definitions were different for each year of input data.

Lastly, for a small number of indicators (Indicators 4B and 6B), it was necessary to use predecessor wards to fill missing data where input data were given by ward definitions that had been superseded. A “predecessor ward” is any ward which, due to boundary changes or any redefinition, was superseded by ward definitions for the target year (i.e. 2022). This was not possible using standard ONS look-ups. Instead, bespoke year-to-year mappings were created by the authors using the ONS master change history, which contains all ward code and name definitions and changes since 1961^52^. A consistent process was used such that the degree of missingness before and after predecessor filling was quantified to verify the effect of this filling.

**Fig. 2:**
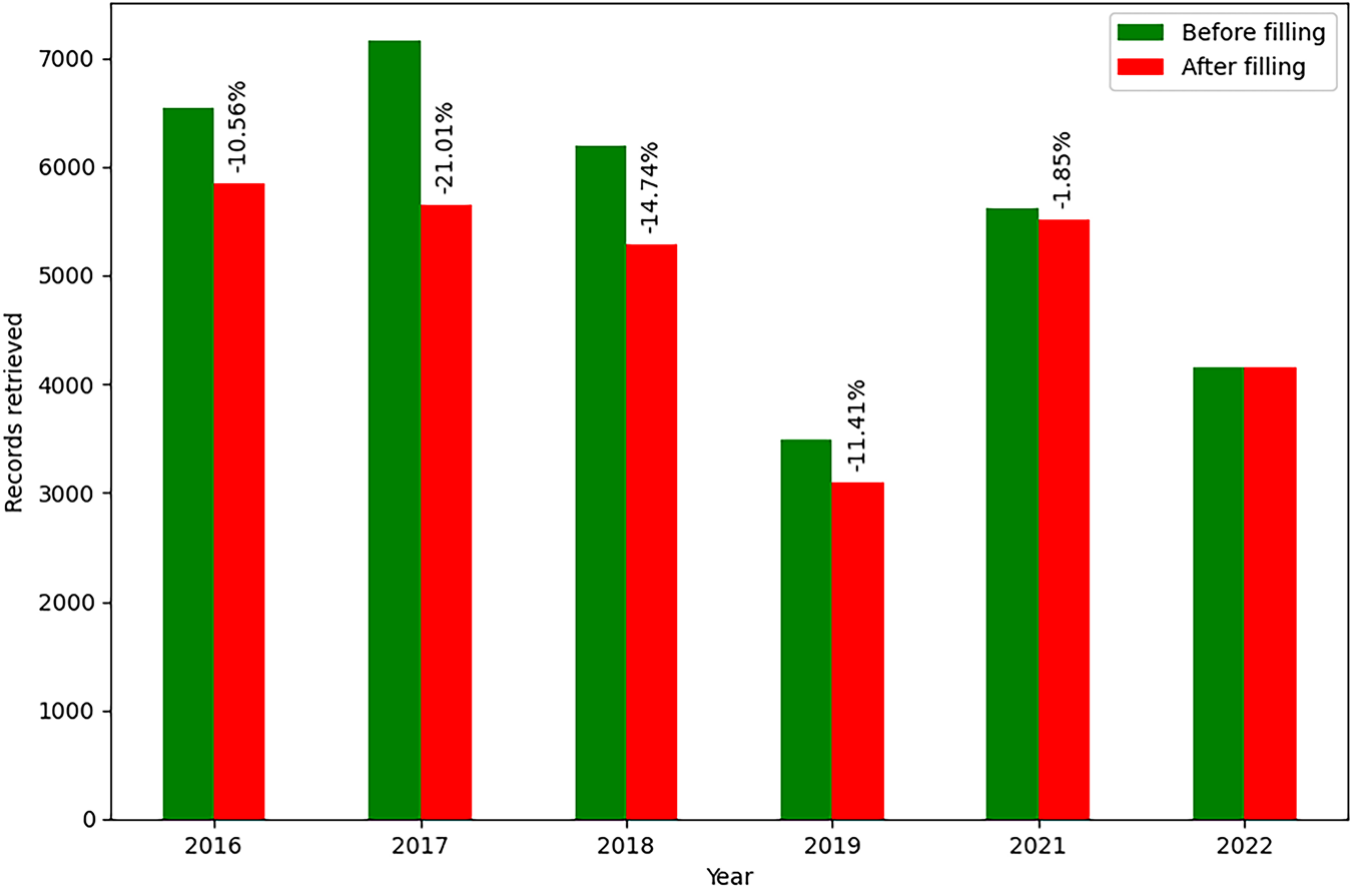
Effect of processor filling of records for Indicator 6B. Percentage value above bars is relative reduction in missingness after predecessor filling. Target year is 2022, so no filling was necessary for that year (see main text).

To illustrate the process of predecessor filling, its effect on the final metric for Indicator 6B is shown in Fig. 2. As indicated in the figure, this filling reduced missingness significantly (e.g. by more than 20% for 2017 input data). The process for Indicator 6B was as follows: for each year of input data (i.e. 2016–2022, excluding 2020), a year-to-year map was constructed (e.g. 2016 to 2022, etc.) from the ONS change history, and a set of predecessor wards for each 2022 ward was identified. For every 2022 ward for which predecessors were thus identified, and if no data existed for that ward, then the data for all predecessor wards were aggregated (by simple mean) and applied to the 2022 ward. A similar process was used for Indicator 4B, the input data for which were given by 2020 wards (England and Wales); missingness was thereby reduced from 27% to 0.6–0.7% for 2019–2021 for that indicator.

**Fig. 3:**
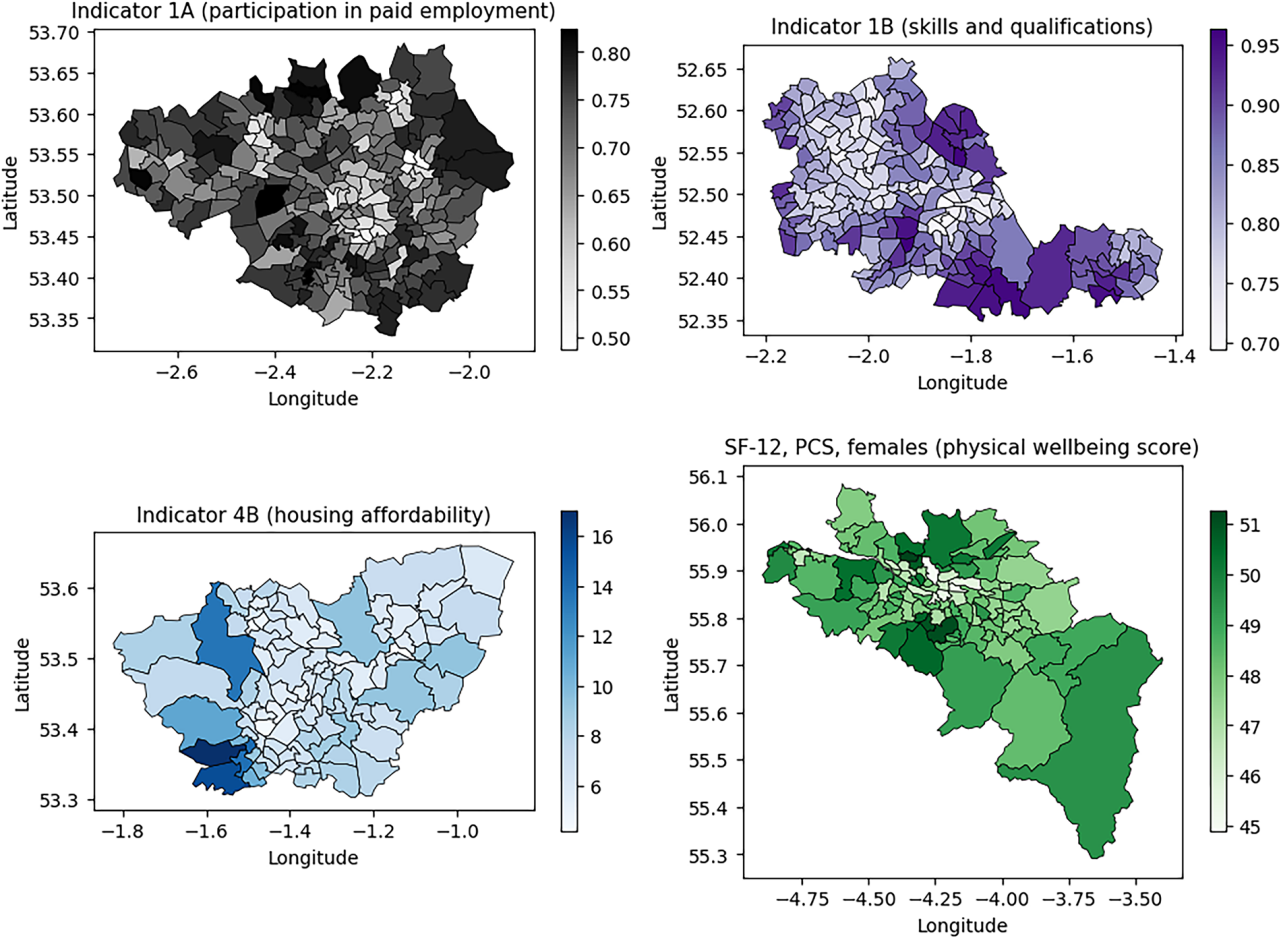
Some indicators at electoral ward level in four city regions. Clockwise from top left: Greater Manchester Combined Authority (Indicator 1A, participation in paid employment), West Midlands Combined Authority (Indicator 1B, skills and qualifications), Glasgow City Region (SF-12 PCS physical health component score, females only) and South Yorkshire Combined Authority (Indicator 4B, housing affordability).

In addition to the 13 inclusive economy indicators, the dataset includes a range of health and supplementary demographic indicators. The health indicators (i.e. SF-12 scores) were derived primarily from the SIPHER Synthetic Population. All demographic indicators (sex ratio and total dependency ratio) were calculated from ONS data.

### Imputation of missing data

Following the predecessor filling process described earlier, and a “rolling” of values from adjacent years (where possible and justified), the average level of missingness across all indicators for the period 2019–2021 was found to be low, at 0.3% overall, and highest for Indicator 6B, at 1.55%. One key factor contributing to this low level of missingness was the use of synthetic data for a substantial number of indicators.

Overall, there are several potential sources of missingness. For example, data might simply not be present (e.g. Indicator 6B, as local elections are only held in certain areas in certain years), data might have been omitted from input sources for reasons of sparsity/confidentiality (in the case of very small or sparsely populated areas such as the City of London and the Isles of Scilly), or missingness might result from errors in the input data. To maximise the utility of the dataset for other modelling and research, a comprehensive data imputation process was performed to reduce the level of missingness across all indicators to zero. The *Amelia II* package^53^ was used for this purpose. The package employs a Bayesian multiple imputation algorithm. There is a diverse range of approaches to the imputation of missing data^54^, and *Amelia II*^53^ contrasts simpler mean-based and regression-based approaches which often risk mis-specifying complex (often non-linear) relationships appropriately. We chose *Amelia II* for two overarching reasons – mainly its methodological advantage and to ensure process consistency. Firstly, from a methodological perspective, *Amelia II* is well-suited to deal with the inherent time-series and cluster character of observations in our data; allows for the specification of auxiliary information; and runs in parallel by default, which optimises performance in large-scale data settings. Combined, these factors equipped *Amelia II* with a slight advantage over other approaches of similar complexity, such as those specified in MICE^55^ as well as other machine learning-based approaches. Secondly, we aimed to ensure a maximum amount of process consistency in relation to the local authority-level dataset^24^, in which we also used *Amelia II*.

Missing values across all indicators (after rolling) were imputed for the period 2019–2021, i.e. the 13 inclusive economy indicators and the demographic and health indicators. The imputation algorithm was run 1,000 times, from which median values were computed as all variables were strictly continuous. Our data rolling strategy is described per indicator in Table 4.

## Data Records

Our dataset consists of 13 inclusive economy indicators (1A, 2A, …, 1B, 2B, …, etc.), two demographic indicators (total dependency ratio, TDR, and sex ratio, SR) and two health indicators (age-standardised SF-12 mental and physical component scores, hereafter SF-12 MCS and PCS; separately for males and females) for 7,973 of GB’s 8,020 electoral wards (2022 definitions), where data for 47 wards are missing due to small population, etc. The dataset covers three years: 2019, 2020 and 2021. In addition, the inclusive economy indicators are in two categories: those associated with economic outcomes (category A) and those associated with wider outcomes and enablers (category B)^8^. A summary of all variables present in the dataset are given in Table 5, and a view of the dataset is given in Fig. 8.

**Table 5:** Summary of variables as they appear in the dataset.

Variable name in dataset	Variable ID (see Table 1, Table 2 and Table 3)
WD22CD	ONS ward code, 2022 definition
WD22NM	ONS ward name, 2022 definition (English)
LAD22CD	ONS local authority code, 2022 definition
LAD22NM	ONS local authority name, 2022 definition (English)
RGN22CD	ONS region code, 2022 definition
RGN22NM	ONS region name, 2022 definition (English)
pop	Population of LSOA/DZ
year	Year
indicator_1a	Indicator 1A, participation in paid employment*
indicator_2a	Indicator 2A, involuntary exclusion from the labour market*
indicator_3a	Indicator 3A, wealth inequality*
indicator_4a	Indicator 4A, earnings inequality*
indicator_5a	Indicator 5A, poverty*
indicator_6a	Indicator 6A, decent pay*
indicator_7a	Indicator 7A, job security*
indicator_1b	Indicator 1B, skills and qualifications**
indicator_2b	Indicator 2B, digital connectivity**
indicator_3b	Indicator 3B, physical connectivity**
indicator_4b	Indicator 4B, housing affordability**
indicator_5b	Indicator 5B, cost of living**
indicator_6b	Indicator 6B, inclusion in decision-making**
tdr	TDR, total dependency ratio***
sr	SR, sex ratio***
sf12mcs_male	SF-12 MCS (mental component score), males only***
sf12mcs_female	SF-12 MCS (mental component score), females only***
sf12pcs_male	SF-12 PCS (physical component score), males only***
sf12pcs_female	SF-12 PCS (physical component score), females only***
sf12mcs	SF-12 MCS (mental component score), males and females***
sf12pcs	SF-12 PCS (physical component score), males and females***

*See Table 1

**See Table 2.

***See Table 3.

The dataset is available as an open-access resource via the Open Science Framework (OSF)^35^, which includes the code used to construct the dataset and detailed instructions for replication. The data repository is intuitively structured, as detailed below.

- Top level folder. This contains the final version of our dataset (“SIPHER Inclusive Economy (Ward Level) Dataset.csv”), along with the user guide.
- Per-indicator folders. Each indicator has a separate folder containing (where applicable): input data, code to produce the final indicator, and the processed data to be aggregated into the full dataset. These can be ignored by the general user, although users can inspect input data and replicate these.
- Compiled output folder. These are files produced during the generation of the dataset, including an unimputed version of the dataset.
- Persistent data and utilities folder. This folder contains any reference data used during generation of the dataset, including text files detailing sources.
- Visualisation folder. Everything required to run some visualisation tools is here, and detailed instructions for doing so are given in the user guide.

## Technical Validation

### Overall internal validation strategy

A summary of the steps taken for internal data validation is given in Table 4, which also contains a summary of all issues found with input data during development, and a quantification of missingness, including before *and* after any data remediation. Table 4 also contains a list of the years for which input data could be found, as the availability of input data varied between indicators, as well as any data rolling that was performed – for the purpose of data imputation – where data were missing for certain years. Also included are details of any area-to-area or year-to-year mappings that were performed due to input data not being available in the correct format (i.e. 2022 electoral wards), or where it was necessary to aggregate from LSOA to ward level.

We used a single set of standard ONS look-up sources for LSOAs/DZs, wards and local authorities throughout, wherever possible, except for predecessor filling for Indicators 4B and 6B, for which the ONS master change history^52^ was used.

In addition to the information provided in Table 4, more comprehensive checks were carried out for each indicator. First, missing or invalid data were replaced with a consistent numerical format to ensure they were ignored during calculations elsewhere in the data generation pipeline. Second, manual spot checks were performed for all indicators, covering the entire data pipeline, i.e. from input data, through any intermediate variables and aggregations, to final metrics.

### External validation: comparison to Indices of multiple deprivation by rank

It is clear from the results presented by Wu, *et al*.^39^ that a synthetic population approach can give detailed insights into area-level health and health inequalities, specifically in that study subjective wellbeing and SF-12 PCS and MCS scores at LSOA level in various UK city regions. To verify that the dataset presented here can provide reliable insights into economic inclusion and other health-related indicators at a granular spatial resolution, we compared multiple indicators to IMD for England, Scotland and Wales.

Typically, the IMD consist of multiple sets of indicators/scores and rankings by LSOA (England and Wales) or DZ (Scotland) and are presented for multiple domains corresponding to broad aspects of people’s lives, e.g. employment, income, employment and health. As highlighted earlier, separate datasets are produced for England, Scotland and Wales^56–58^. The IMD are intended to “provide a set of relative measures of deprivation for small geographical areas”^56^ (p. 7) and allow the degree and nature of deprivation amongst small areas to be compared in a flexible way.

**Fig. 4:**
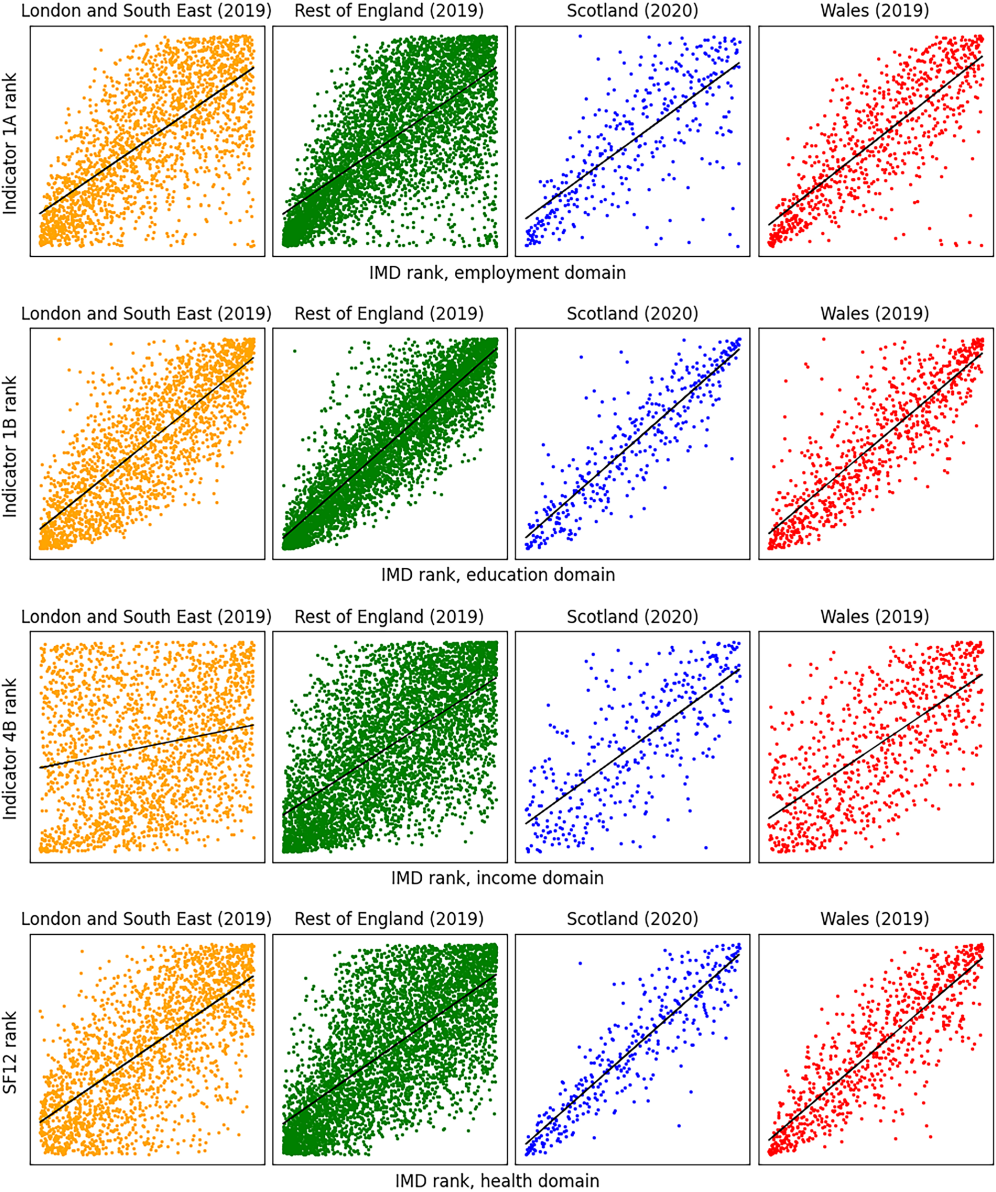
Comparison of IMD domains to specific indicators at electoral ward level. From top to bottom: employment domain (IMD) and Indicator 1A (participation in paid employment; this dataset), education domain and Indicator 1B (skills and qualifications), income domain and Indicator 4B (housing affordability) and health domain and SF-12 (wellbeing score, both sexes, PCS and MCS combined). See main text for methodology of aggregation of IMD data from LSOA/DZ to ward level. Solid black lines: linear best fit.

For validation of our dataset against external references, four IMD domains were compared to four specific indicators for which the respective definitions were most similar. Although domain-indicator comparator sets were selected that were as similar as possible, the exact variables and metrics differed in detail in all cases. As noted by James, *et al*.^59^, choosing comparators in this way results in “a minimal risk of circularity when exploring relationships” (p. 9). The comparisons are shown in Fig. 4 for the latest versions of the IMD datasets that are available, i.e. 2019 for England and Wales, and 2020 for Scotland; data for those years for each indicator were also used. It is also noted that England is disaggregated into two super-regions in Fig. 4(a) London and the southeast of England, and (b) the rest of England – to explore differences between those super-regions.

IMD data are generally provided in the form of scores and ranks. Because conversion between IMD scores and inclusive economy indicator values would be very complex, comparisons were made by rank. IMD data are provided at LSOA/DZ level, so it was necessary to aggregate to electoral ward level for comparison with the 13 inclusive economy indicators. To do so, the methodology described in a recent report by the Greater London Authority^60^ was followed, specifically the “rank of average ranks” method. Mid-year population estimates by LSOA were used for aggregation to electoral ward. However, population estimates for 2021 are not available for 2011 LSOAs as used throughout this study, so 2020 population estimates were carried forward to 2021 (England and Wales only). Population estimates are from the ONS data service Nomis for England and Wales^61^, and from the Scottish Government statistics service for Scotland^62^.

For all four comparator sets, the correlation is generally good, particularly for the education domain/Indicator 1B comparison. This is not surprising, as the metric for Indicator 1B incorporates the level of educational attainment of adults in each area, while all versions of the IMD education domain also include an indicator relating to the level of education of adults in each area. However, corresponding IMD indicators also capture other concepts relating to child and youth educational attendance, enrolment or attainment. The correlation is less good for the income domain/Indicator 4B comparison, particularly for London and the southeast of England. This is also not surprising, as Indicator 4B is a composite of house prices and individual incomes, whereas all versions of the IMD income domain are comprised mainly of various rates of receipt of income-related benefits. Therefore, the definitions of metrics differ significantly, and the influence of house prices – which are particularly high in the south of England – is not represented in the IMD data.

### External validation: comparison to local authority-level dataset

We compared the 13 inclusive economy indicators in our dataset to the corresponding ones in the SIPHER Inclusive Economy (Local Authority Level) dataset, as reported by Lomax, *et al*.^24^ and used by Höhn, *et al*.^25^. The comparisons were reviewed, and several examples are shown. Data in all comparisons were aggregated to regional level to compare like with like. The same method of aggregation as was used for comparison to the IMD by rank (in that case from LSOA to region) was used here for comparison by value, i.e. population-weighted means using small-area population estimates from the same sources as the previous section. It is noted that the full set of 13 comparisons are not shown here, for brevity.

Of the 13 indicators, some showed extremely good agreement and others less good. Several factors might account for the observed differences. The first factor to consider is similarity – or lack of it – between exact indicator definitions (for example, Indicator 5A, poverty). The second factor is similarity with respect to the data sources used for each indicator, which differed between the two datasets for some indicators (for example, Indicator 5B, cost of living). The third factor is the numerical nature of indicators themselves and their suitability – or otherwise – for aggregation to regional level (for example, Indicator 3A, wealth inequality; and Indicator 4B, housing affordability).

**Fig. 5:**
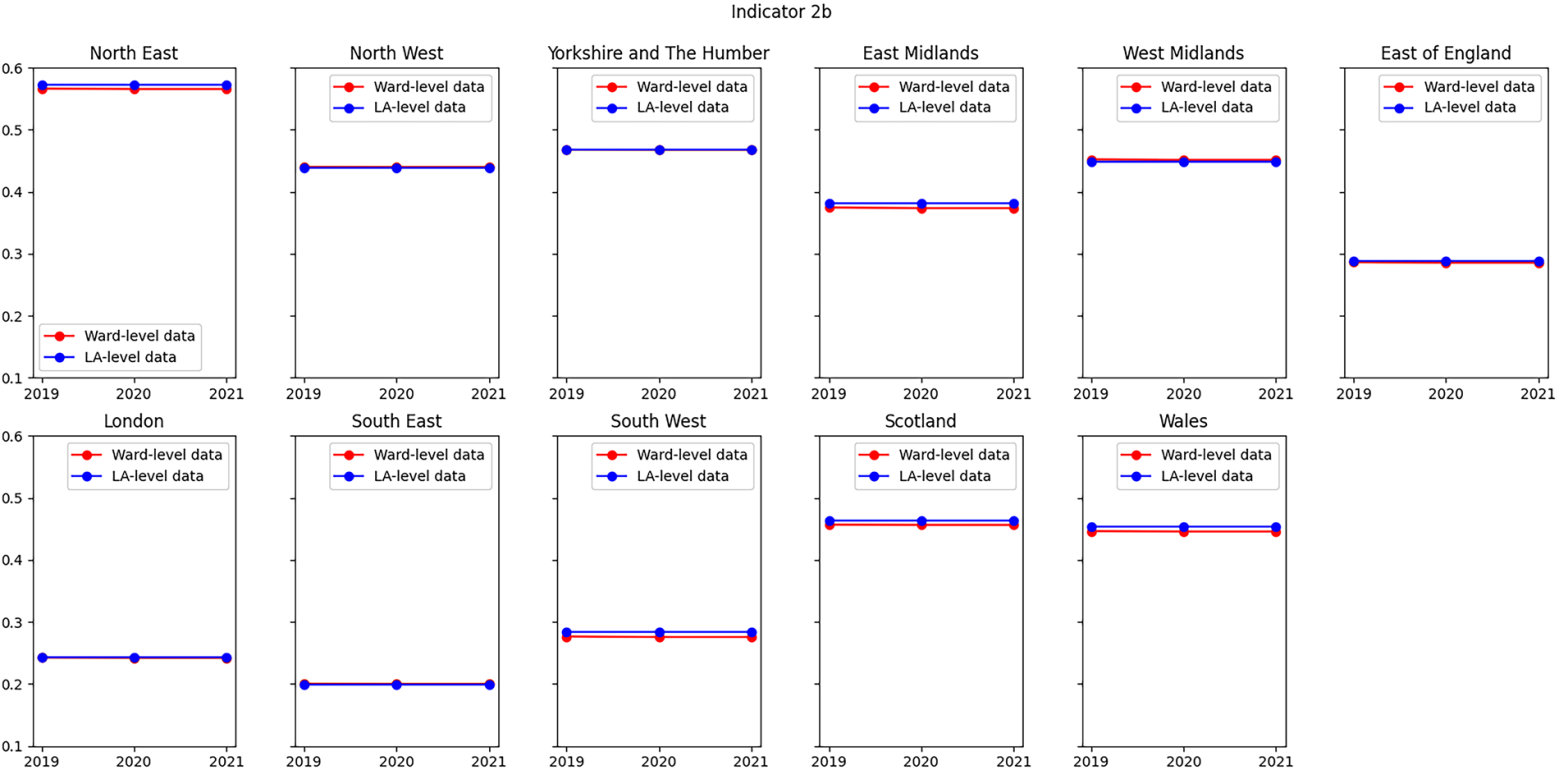
Comparison of Indicator 2B (digital connectivity) from this dataset to that in SIPHER Inclusive Economy (Local Authority Level) dataset at regional level. Metric in both datasets is proportion of LSOAs within ward or local authority that are digitally disengaged (see Table 2).

Of those comparisons that agreed particularly well (Indicators 1A, 2A, 7A, 2B, 3B, 4B and 6B), an example is shown in Fig. 5 for Indicator 2B (digital connectivity). The agreement is excellent, which is to be expected since (a) exactly the same metric is used, albeit aggregated to different geographical scales, (b) the underlying data source is the same for both the ward- and local authority-level datasets (the primary data source is at LSOA level) and, (c) the numerical nature of the metric is entirely suitable for aggregation to larger geographies.

**Fig. 6:**
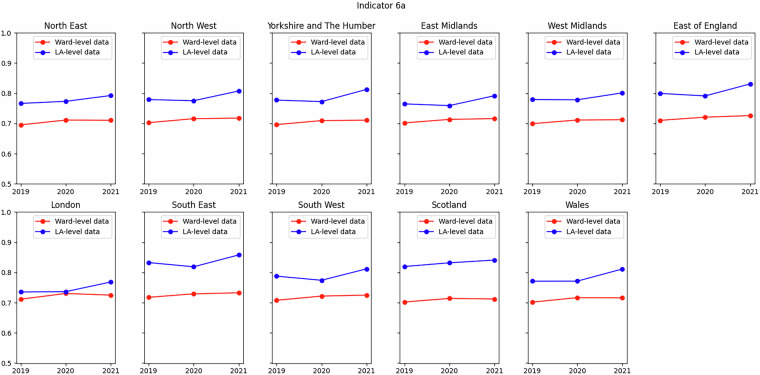
Comparison of Indicator 6A (decent pay) from this dataset to that in SIPHER Inclusive Economy (Local Authority Level) dataset at regional level. Metric in ward-level dataset is proportion of employee jobs paid at or above the Real Living Wage, metric in local authority-level dataset is proportion of employee jobs paid at or above the National Living Wage (see Table 1).

Of those indicators that compared well (Indicators 3A, 4A, 6A and 1B) but not as well as those described above, an example is shown in Fig. 6 for Indicator 6A (decent pay). The difference between the two datasets is of the order of a few percent. Nevertheless, the agreement is striking and remarkable, given that the data sources differ greatly: the Annual Survey of Hours and Earnings in the local authority-level dataset, and multiple UKHLS variables obtained from the synthetic population, aggregated from LSOAs/DZs for the ward-level dataset.

**Fig. 7:**
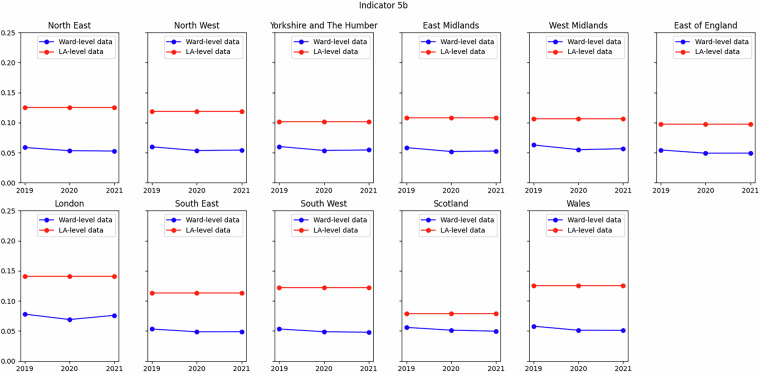
Comparison of Indicator 5B (cost of living) from this dataset to that in SIPHER Inclusive Economy (Local Authority Level) dataset at regional level. Metric in ward-level dataset is proportion of household experiencing fuel poverty, metric in local authority-level dataset is proportion of households experiencing food insecurity (see Table 2).

Of those indicators that compared less well (Indicators 5A and 5B), an example is shown in Fig. 7 for Indicator 5B (cost of living). The difference between the two datasets for this indicator can entirely be accounted for by the definition of metrics used in each case. In the local authority-level dataset the metric is the extent of food insecurity, whereas in the ward-level dataset the metric is the proportion of adults living in fuel-poor household, as measured by the UKHLS variable *hheat*. For Indicator 5A, the local authority-level dataset uses the rate of child poverty after housing costs (AHC), whereas this dataset uses the rate before housing costs (BHC), as data for the former are not available at ward level.

### Final comments

The SIPHER Inclusive Economy (Ward Level) dataset is presented as an all-GB, harmonised resource and is intended as a contribution to address the scarcity of small-area datasets focused on economic inclusion and its relationship with health and health inequality. It is intended for use by researchers, stakeholders and policy-makers where small-area variation exists – for example, *within* larger administrative areas such as local authorities or (city) regions – that require commensurate small-area actions. A comprehensive, consistent data sourcing and validation process was followed, and several indicators were compared to external datasets, including the Indices of Multiple Deprivation, demonstrating that the dataset correlates well with existing data sources, without duplicating them in terms of metrics. The dataset is therefore also intended for researchers seeking all-GB area rankings.

It is noted that the inclusive economy indicators presented in the dataset, if updated appropriately, can be used to track and assess the effect of policies at small spatial resolution. From a longitudinal perspective, several aspects of economic inclusion, as defined earlier^9,13^ can be monitored, i.e. deliberate design of an economy to be inclusive; equitable distribution of economic benefits; and equitable access to resources necessary for economic participation. Broadly, these three aspects of economic inclusion can be linked to different indicators in the dataset, allowing some part of the complexities of people’s everyday lives to be quantified. As well as several examples of quantification of economic inclusion and health inequality over time, spatial visualisations – both here in the form of city region-level plots of various indicators, and through the online visualisation tool – are provided to aid decision-making.

## Usage Notes

### Application across policy and research

**Fig. 8:**
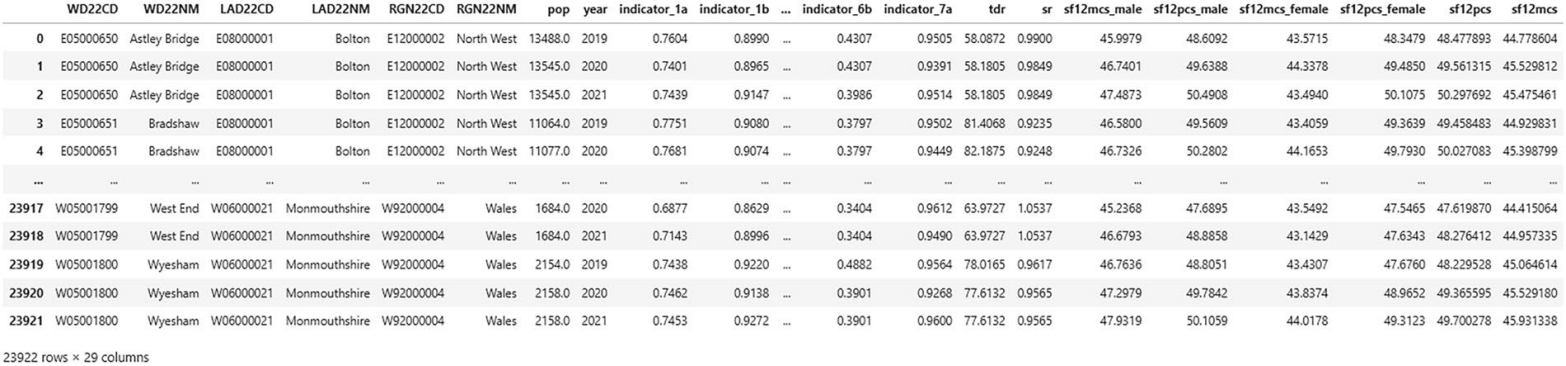
A view of the dataset, taken from the visualisation tool available in the online data repository. Indicators 1B to 5B not shown.

The dataset is presented at the granular spatial level of electoral wards according to the 2022 boundaries. A view of the structure of the dataset is given in Fig. 8, which was created using the visualisation tools provided at the data repository^35^.

As a result of the fine spatial scale of the dataset, variation within higher-level geographies such as local authorities or city regions can easily be visualised. To follow the format of visualisations given by James, *et al*.^59^ and Wu, *et al*.^39^, the distribution of indicator values for four city regions are presented in Fig. 3, specifically Greater Manchester, West Midlands Combined Authority, Glasgow City Region and South Yorkshire Combined Authority. This example illustrates the opportunities for spatial analysis and visualisations arising from the dataset (spatial boundaries of 2022 wards are from the ONS^63^).

To support swift data exploration, a web-based resource has been developed in the form of an interactive data visualisation tool (https://mapmaker.cdrc.ac.uk/#/inclusive-economy/). It provides a code-free approach to exploring the dataset, and the geographical distribution of the indicators can be viewed flexibly.

### Updateability of the dataset

Updateability of granular spatial data is an important factor considered by policy-makers^14^. Our dataset was conceptualised with updateability in mind, e.g. as more recent data become available. However, the ease with which a dataset such as the one presented here can be updated varies according to the nature and availability of the data on which it is based, and so the effort required to update the indicators would vary.

In our dataset, most of the variables derived from the SIPHER Synthetic Population can be updated easily, since new waves of survey data are generally made available annually. Updating the SIPHER Synthetic Population itself, though outside the remit of this study, requires a much more involved process but is generally possible. Updating variables from non-synthetic sources – for example, national statistical agencies – is straightforward only if new data are released regularly. However, this is not always the case. For example, the ONS’ House Price Statistics for Small Areas in England and Wales (HPSSA) data series, which was used for Indicators 3A and 4B, was discontinued in the format used here in 2023, and would therefore need to be replaced with another data source going forward. In addition, some of the input data used here are from bespoke sources – i.e. Indicators 2B (bus accessibility data) and 3B (IUC data) – and are unlikely to be updated.

Overall, external data sources – such as those used for Indicators 2B and 3B – present the most challenges in terms of updateability. However, on the general case of updating the dataset presented here to more recent boundary definitions, we note that it is possible to account for boundary changes post hoc using best-fit and portioning approaches based on mapping and look-up tables, which are routinely published by the ONS (for England and Wales) and the Scottish Government.

## Data Availability

All code used and data generated or analysed during this study are available via a data repository^35^, including a user guide on how to use the dataset and create some visualisations of it using the Python notebook provided.
